# Prevalence of Sexual Victimization and Correlates of Forced Sex in Japanese Men Who Have Sex with Men

**DOI:** 10.1371/journal.pone.0095675

**Published:** 2014-05-06

**Authors:** Yasuharu Hidaka, Don Operario, Hiroyuki Tsuji, Mie Takenaka, Hirokazu Kimura, Mitsuhiro Kamakura, Seiichi Ichikawa

**Affiliations:** 1 Takarazuka University School of Nursing, Osaka city, Osaka, Japan; 2 Brown University School of Public Health, Providence, Rhode Island, United States of America; 3 Japanese Foundation for AIDS Prevention, Osaka city, Osaka, Japan; 4 Kansai AIDS Council, Osaka city, Osaka, Japan; 5 Health and Social Welfare Bureau, City of Yokohama, Yokohama city, Kanagawa, Japan; 6 Keio University Graduate School of Health Management, Fujisawa city, Kanagawa, Japan; 7 Nagoya City University School of Nursing, Nagoya city, Aichi, Japan; Rollins School of Public Health, United States of America

## Abstract

Studies of men who have sex with men (MSM) in diverse geographic and cultural contexts have identified health challenges affecting this population. MSM might be particularly vulnerable to sexual victimization and forced sex. The aim of this research study was to examine prevalence of sexual victimization and correlates of forced sex among Japanese MSM. We recruited a sample of 5,731 Japanese MSM who completed an internet-administered survey. Participants reported on history of different types of sexual victimization, unprotected anal sex, other health risk behaviors, exposure to gay-related teasing and bullying, depression, and suicidality. Over one-fifth of the sample (21.4%) reported experiencing at least one form of sexual victimization, and 8.7% reported a history of forced sex. MSM who had ever experienced forced sex were significantly more likely to report experiencing psychological risks (depression OR = 1.55, 95% CI = 1.28–1.89; attempted suicide OR = 2.25, 95% CI = 1.81–2.81; other forms of bullying OR = 1.38, 95% CI = 1.13–1.68) and other behavioral risks (unprotected anal sex OR = 1.56, 95% CI = 1.29–1.90; sex venue attendance OR = 1.27, 95% CI = 1.04–1.54; methamphetamine use OR = 1.57, 95% CI  = 1.05–1.36), compared to MSM who had not experienced forced sex. Efforts to develop holistic and integrated health services for Japanese MSM are warranted, particularly related to psychosocial determinants of HIV prevention. However, due to cultural factors that emphasize familial and social relations and that stigmatize same-sex behavior, Japanese MSM might experience challenges to seeking social support and health services. Interventions must be provided in safe and non-judgmental settings where Japanese MSM feel comfortable disclosing their health and social support needs.

## Introduction

Globally, there have been an increasing number of studies examining health and psychosocial risk factors affecting men who have sex with men (MSM) [1]. Much of this attention has focused on disproportionate HIV prevalence among MSM across international settings [2]–[4]. However, additional research has also shown that MSM in diverse geographic regions may also experience psychological and social vulnerabilities – such as discrimination and interpersonal violence – which can contribute to further health challenges in this population [5]–[9]. A nascent literature has examined health outcomes in Japanese MSM. Some of the documented health challenges among Japanese MSM include HIV risk [10], drug use [11], and suicidal ideation and attempted suicide [12]. Studies to date suggest a need for enhanced understanding into the risk factors for health problems in Japanese MSM.

Previous research has suggested that adverse behavioral and psychosocial health indicators in Japanese MSM might be due, in part, to exposure to social stressors [13]. For example, stigma, homophobic abuse, and victimization are forms of social stress reported in MSM samples in Japan [12], [13]. Experience of homophobic stigma has been shown to be related to psychological problems and sexual risk behaviors in MSM populations in other parts of Asia [14]–[17]. This finding is consistent with minority stress theory [18], which postulates that exposure to negative social or interpersonal events can compromise the psychological well-being of sexual minority individuals and thereby contribute to higher prevalence of mental and physical health problems in MSM.

Sexual victimization is an extreme form of social and interpersonal stress that MSM may experience, and can contribute to further psychological and behavioral health risks among those who have been victimized. Sexual victimization can be defined as any form of involuntary sexual interaction or contact with another person, which can occur in childhood as well as in adulthood [19]. Studies have shown that MSM with a history of childhood sexual victimization show greater sexual risk behavior in adulthood and have higher prevalence of HIV infection compared with their MSM peers who have not experienced sexual victimization [20]. There are few studies that have examined adult sexual victimization experiences among men. This may be due, in part, to underreporting of adult sexual victimization, stigma about discussion of sexual victimization in adult men, and myths about men's vulnerability to sexual victimization [21]. However, a review of research on sexual victimization in adult men found that MSM were more likely to report experiences of adult sexual victimization compared with heterosexual men [21]. Forced sex is a specific type of sexual victimization that has extremely adverse physical and mental health consequences among male victims. Individuals with a history of forced sex can have long-term risk for HIV, trauma, and maladaptive health risk behaviors [22].

To date, there are no known studies exploring sexual victimization, including forced sex, among Japanese MSM and its potential role in affecting the health and psychological well-being in this population. To enhance understandings of the health of Japanese MSM, the aims of this paper are to explore (i) the prevalence of different types of sexual victimization in a large population sample, and (ii) associations between history of forced sex and other psychosocial and behavioral risk factors. Because this is an understudied topic, findings from this analysis can provide insight for future research and potentially guide interventions to address the health and well-being of MSM in Japan who have a history of sexual victimization and forced sex.

## Method

### Recruitment

The internet was used to recruit a diverse sample of Japanese MSM for a study of health behaviors and well-being. The internet has been argued to be an acceptable method for collecting large, heterogeneous samples of hard-to-reach populations [23], [24]. Internet technology can be helpful in reaching gay, bisexual, and questioning men who are less comfortable attending homosexual-themed venues, such as bars and nightclubs. Data collection through the internet can also increase the opportunity for participants to respond anonymously by avoiding face-to-face contact [11], [13], which might be a barrier to participation due to MSM stigma in Japanese culture. Informational announcements about the study were placed on internet websites catering to Japanese MSM audiences. In addition to posting banners on gay-related websites, recruitment strategies included: flyers distributed in gay venues, announcements posted in gay organizational newsletters as well as in gay magazines, and announcements posted at social network websites catering to gay men. We designed the internet banners and informational flyers in a manner that would draw the attention of MSM, e.g., using physically attractive male models. However, we designed a range of recruitment media (e.g., information-only announcements without pictures or using gay-relevant slogans and symbols) to minimize bias associated with recruiting men solely based on their response to sexually suggestive images. Announcements provided information about the research project and eligibility for participation. Potential participants were directed to an internet site to learn more about the study. Participant inclusion criteria included: 1) being a Japanese male who has ever had sex with men; 2) having internet access; 3) having Japanese written language fluency.

### Procedure

Participants who met inclusion criteria entered a secured internet website to complete the anonymous online survey. The website first presented informed consent information. If participants understood the purposes of the study and agreed to the terms of participation, they clicked an “Agree” button, and they then accessed the questionnaire. All items and response options were presented in Japanese language, and participants' responses were immediately saved in a firewall-protected database. To minimize the chances that participants would complete the survey multiple times, we examined internet protocol addresses and internet providers encoded within the data and, if encoded information appeared similar, checked the demographic data for redundant information. Internet protocol addresses were deleted before conducting analysis. Using a procedure validated previously in an internet study of MSM in Japan, we asked participants to define two terms that were identified through earlier formative research as well-known colloquial expressions in the Japanese MSM/gay community (which translated into English would mean “gay men/gay society” and “heterosexual”) [11], [13]. Data from men who were unable to define the terms were excluded from analysis. Data were collected between August 11 and November 30, 2005. The study protocol was approved by the Ethics Committee of Nagoya City University School of Nursing.

In total, 6,260 participants attempted to complete the questionnaire, 196 people were excluded due to missing data, 140 were excluded because of data duplication or because they could not define the slang terms, 73 were excluded because they were not males, and 120 were excluded because they did not live in Japan.

### Measures

Participants reported demographic characteristics, including age, highest educational level, and sexual orientation (gay, bisexual, heterosexual, undecided, unsure and other). Participants were asked whether they had ever experienced a range of sexual interactions *against their will*, including the following: being undressed by another person, target of verbal sexual abuse, forced to kiss another person, sexually touched by another person, forced to touch the genitals of another person, forced to engage in vaginal sex with a female, forced to engage in oral sex (with a male or female), forced to engage in anal sex with a male, and any other form of unwanted sexual interaction. Participants also described whether they had ever been harassed or bullied in school by others due to their sexuality, and whether they had close gay/bisexual friends to whom they could confide in and close heterosexual friends to whom they could confide in. They completed the Japanese version of the Center for Epidemiologic Studies Depression Scale (CES-D); participants were categorized as being moderately depressed based on a score greater than 16 [25]. Participants also reported whether they had ever attempted suicide. Finally, participants reported their HIV status, frequency of condom use when engaging in anal sex, and recent methamphetamine use.

### Data analysis

Statistical analysis was conducted using SPSS v.21. First, we described the prevalence of different types of sexual victimization, sociodemographic variables, and self-reported psychosocial and behavioral health variables. Second, we examined correlates of forced sex, using chi-square tests to assess bivariate associations and multivariable logistic regression to identify independent associations controlling for other co-variates. Multivariable regression analysis was based on procedures described in Hosmer & Lemeshow [26], in which we entered into the regression model any variable that had a moderate bivariate association with forced sex at *p*<.25. Adjusted odds ratios (ORs) and 95% confidence intervals (95% CIs) were reported.

## Results

Data from a total of 5,731 respondents are included in this analysis (Table 1). The mean age was 30.8 years (SD = 8.9, range = 12–82), with 6.5% between 12 and 19 years old, 42.4% between 20 and 29 years old, 35.5% between 30 and 39 years old, 11.4% between 40 and 49 years old, and 3.6% over the age of 50 (n = 34 did not report their age). Over half (56.4%) of the sample had completed a University degree. Over two-thirds (67.5%) identified themselves as gay and 25.9% identified as bisexual. Over half (54.5%) had been verbally teased with words such as “homosexual, faggot, fag” and 45.1% had experienced other forms of bullying. The majority of participants reported having close gay/bisexual male friends (65.1%) as well as close heterosexual friends (58.6%). Over one-third (38.8%) reported moderate levels of depression (CES-D >16), and 14.0% had ever attempted suicide. Overall, 5.3% identified as HIV-positive, 48.7% reported having unprotected anal sex in the past six months, 52.6% had visited a sex venue in the past six months, and 3.7% had ever used methamphetamines.

**Table 1:** Participant characteristics and associations with history of forced sex in a sample of Japanese MSM (n = 5,731).

		Total		Lifetime experience of forced sex		
		n	%	n	%	*p-value*
Overall		5,731		500	8.7	
Age group	12–19	371	6.5	36	9.7	0.002
	20–29	2,432	42.4	253	10.4	
	30–39	2,037	35.5	149	7.3	
	40–49	652	11.4	48	7.4	
	50+	205	3.5	11	5.4	
	Missing	34	0.6	3	8.8	
Sexual orientation	Gay	3,868	67.5	337	8.7	0.25
	Bisexual	1,484	25.9	138	9.3	
	Other	379	6.6	25	6.6	
Educational level	No university degree	2,496	43.6	236	9.5	0.089
	University degree	3,235	56.4	264	8.2	
Ever been teased verbally with words such as “homosexual, faggot, fag”	No	2,605	45.5	193	7.4	0.001
	Yes	3,126	54.5	307	9.8	
Ever been bullied other than verbal teasing	No	3,146	54.9	220	7	<0.001
	Yes	2,585	45.1	280	10.8	
Depression in past week	Not depressed	3,510	61.2	235	6.7	<0.001
	Depressed	2,221	38.8	265	11.9	
Ever attempted suicide	No	4,926	86	358	7.3	<0.001
	Yes	805	14	142	17.6	
Went to any sex venues in 6 months	No	2,714	47.4	205	7.6	0.003
	Yes	3,017	52.6	295	9.8	
Have close gay/bisexual friends	No	2,000	34.9	156	7.8	0.069
	Yes	3,731	65.1	344	9.2	
Have close heterosexual friends	No	2,370	4.4	175	7.4	0.003
	Yes	3,361	58.6	325	9.7	
HIV status	Negative	5,425	94.7	457	8.4	0.002
	Positive	306	5.3	43	14.1	
Ever used methamphetamine	No	5,520	96.3	467	8.5	0.001
	Yes	211	3.7	33	15.6	
Unprotected anal intercourse in 6 months	No	2,941	51.3	198	6.7	<0.001
	Yes	2,790	48.7	302	10.8	

Prevalence of sexual victimization experiences are reported in Table 2. Overall, 21.4% of the sample reported experiencing any of the types of sexual victimization assessed in this survey. The most common forms of sexual victimization included unwanted sexual touching (16.7%), being undressed (10.5%), being forced to kiss someone (9.6%) and being forced to touch someone's genitals (8.8%). A total of 500 participants (8.7% overall; 40.8% of those who reported any sexual victimization) reported ever experiencing any forced sex. Overall, 6.5% experienced forced anal sex, 5.9% experienced forced oral sex, and 2.0% experienced forced vaginal sex.

**Table 2:** Prevalence of different forms of lifetime sexual victimization in a sample of Japanese MSM (n = 5,731).

Forms of sexual victimization	n	%
Undressed	600	10.5
Abused with obscene words	392	6.8
Forced kiss	553	9.6
Touched	957	16.7
Forced to touch genital part	507	8.8
Forced vaginal sex	113	2
Forced oral sex	338	5.9
Forced anal sex	372	6.5
Other	215	38
Any forced sex (vaginal, oral, anal)	500	8.7
Any forms of sexual victimization (any of above)	1,224	21.4

Bivariate correlates of a history of forced sex are listed in Table 1. Forced sex was associated with younger age, experience of verbal teasing due to being gay, experience of other forms of bullying due to being gay, depression, history of attempted suicide, having close heterosexual friends, HIV-positive status, unprotected anal sex, vising a sex venue, and history of methamphetamine use (*p*<.01).

Variables that were independently associated with a history of forced sex are shown in Table 3. Based on multivariable regression analysis, forced sex was shown to be significantly associated with depression (OR = 1.55, 95% CI = 1.28–1.89), history of attempted suicide (OR = 2.25, 95% CI = 1.81–2.81), experience of bullying (OR = 1.38, 95% CI = 1.13–1.68), having close heterosexual friends (OR = 1.26, 95% CI = 1.03–1.55), visiting a sex venue (OR = 1.27, 95% CI = 1.04–1.54), having unprotected anal sex (OR = 1.56, 95% CI = 1.29–1.90), HIV positive status (OR = 1.57, 95% CI = 1.10–2.24), and ever using methamphetamines (OR = 1.57, 95% CI = 1.05–2.36).

**Table 3:** Multivariable regression to identify independent correlates of forced sex in a sample of Japanese MSM (n = 5,731).

		Lifetime experience of forced sex		
		AOR	95% CI	*p-value*
Age group	12–19	ref.		
	20–29	1.03	(0.70–1.51)	0.877
	30–39	0.71	(0.48–1.07)	0.102
	40–49	0.8	(0.50–1.28)	0.354
	50+	0.68	(0.33–1.38)	0.282
	Missing	0.99	(0.28–3.50)	0.983
Sexual orientation	Gay	ref.		
	Bisexual	1.24	(1.00–1.55)	0.055
	Other	0.81	(0.53–1.25)	0.343
Educational level	No university degree	ref.		
	University degree	1.02	(0.84–1.23)	0.865
Ever been teased verbally with words such as “homosexual, faggot, fag”	No	ref.		
	Yes	1.13	(0.93–1.39)	0.229
Ever been bullied other than verbal teasing	No	ref.		
	Yes	1.38	(1.13–1.68)	0.002
Depression in past week	Not depressed	ref.		
	Depressed	1.55	(1.28–1.89)	<.001
Ever attempted suicide	No	ref.		
	Yes	2.25	(1.81–2.81)	<.001
Went to any sex venues in 6 months	No	ref.		
	Yes	1.27	(1.04–1.54)	0.017
Have close gay/bisexual friends	No	ref.		
	Yes	1.12	(0.90–1.38)	0.307
Have close heterosexual friends	No	ref.		
	Yes	1.26	(1.03–1.55)	0.027
HIV status	Negative	ref.		
	Positive	1.57	(1.10–2.24)	0.014
Ever used methamphetamine	No	ref.		
	Yes	1.57	(1.05–2.36)	0.029
Unprotected anal intercourse in 6 months	No	ref.		
	Yes	1.56	(1.29–1.90)	<.001

## Discussion

Over one-fifth (21.4%) of this large sample of MSM in Japan reported experiencing at least one form of sexual victimization as assessed in this study, and 8.7% reported a history of forced sex. MSM who had ever experienced forced sex were significantly more likely to report experiencing psychological risks (i.e., depression, attempted suicide, other forms of bullying) and other behavioral risks (unprotected anal sex, sex venue attendance, methamphetamine use) compared with their peers who did not experience forced sex. These cross-sectional findings suggest that assessing for sexual victimization and addressing the consequences of forced sex might be an important component of clinical screenings or public health interventions related to HIV prevention and mental health services for MSM in Japan.

Findings here are consistent with studies from other settings which indicate that HIV and other health disparities affecting MSM must be understood in the context of psychosocial stressors and contextual factors that determine health risk behaviors among members of this population [18]. Consequently, integrated and holistic approaches to health care for MSM may be warranted – particularly approaches that consider history of adverse psychological and behavioral co-factors that need intervention [1].

Capacity to provide holistic health services to MSM in Japan, however, is currently limited. Among Japanese MSM, 80% have not disclosed their sexual orientation to parents, thus these men may experience difficulty seeking help from their family members. Although poor mental health status such as depression was apparent in this population, experience of accessing mental health services was low [27]. These findings suggest that MSM may experience difficulty seeking support from parents as well as in medical care settings, potentially due to the fear of prejudice and discrimination. Professionals such as mental care providers, nurses, public health professionals providing HIV counseling and testing, and clinical psychologists would benefit from improved training to understand about the needs of this population, in order to provide adequate professional services and support to MSM. Japanese MSM would benefit from resources that identify health service providers or health settings that are friendly and competent in working with sexual minority patients and populations. Currently, there are no known publically available resources to help MSM in Japan identify health services in general, especially mental health care. Development of referral networks, brochures, and websites with information about appropriate and confidential services for MSM is warranted.

There are notable strengths to this study. This is the first known study of the prevalence of sexual victimization and correlates of forced sex in Japanese MSM. Use of the internet allowed us to recruit a large sample of MSM, and suggests the utility of internet and social media for outreach and recruitment to MSM in Japan, a population that might otherwise be hard to reach. Findings expose a need for appropriate and confidential health services for MSM, and suggest the role of sexual victimization as a determinant of behavioral health and psychosocial problems in this population.

Limitations to this study must be considered. First, the study used a cross-sectional design which prevents interpretation of causality or temporal order among variables. Second, although this is a large MSM sample, participants were recruited using non-representative sampling methods. Because we did not use targeted recruitment efforts to achieve a sociodemographically representative sample, findings might not be generalizable to MSM who do not access gay-themed internet or periodical content or men who felt uncomfortable completing an online survey. Third, self-report measures might have been affected by social desirability or recall biases. Fourth, because this was an exploratory study, and the first of its kind in Japan, we did not have access to culturally validated measures of sexual victimization and other risk behaviors in Japanese MSM. Although measures of sexual risk and other health behaviors in this survey have also been reported in previous studies of Japanese MSM [11], [13], future research must better assess the psychometric properties and cultural sensitivity of sexual behavior and victimization measures for use with this population. Fifth, most measures of sexual behavior, victimization, and other risk behaviors in this survey assessed lifetime experience, resulting in limited inferences about temporal windows which might affect health risk.

## Conclusion

In summary, this study highlights the role of prior sexual victimization in contributing to the psychological and behavioral risks of MSM in Japan. Findings reported here correspond with a substantial literature (mostly conducted in the West) on the associations of sexual victimization – including childhood sexual victimization as well as adult victimization – on psychological adjustment and future sexual risk outcomes. Efforts to address these issues among Japanese MSM are warranted. Such efforts must be mindful of cultural and social factors that might challenge provision of holistic services to Japanese MSM, and which might also present barriers to access of health service and disclosure of problems among Japanese MSM.

## References

[1] Mayer KH, Bekker LG, Stall R, Grulich AE, Colfax G, et al (2012) Comprehensive clinical care for men who have sex with men: an integrated approach. Lancet 380: 378–387. 22819653 10.1016/S0140-6736(12)60835-6 PMC5603076

[2] Beyrer C, Baral SD, van Griensven F, Goodreau SM, Chariyalertsak S, et al (2012) Global epidemiology of HIV infection in men who have sex with men. Lancet 380: 367–377. 22819660 10.1016/S0140-6736(12)60821-6 PMC3805037

[3] Smith AD, Tapsoba P, Peshu N, Sanders EJ, Jaffe HW (2009) Men who have sex with men and HIV/AIDS in sub-Saharan Africa. Lancet 374: 416–422. 19616840 10.1016/S0140-6736(09)61118-1

[4] van Griensven F, de Lind van Wijngaarden JW, Baral S, Grulich A (2009) The global epidemic of HIV infection among men who have sex with men. Curr Opin HIV AIDS 4: 300–307. 19532068 10.1097/COH.0b013e32832c3bb3

[5] Adebajo SB, Eluwa GI, Allman D, Myers T, Ahonsi BA (2012) Prevalence of internalized homophobia and HIV associated risks among men who have sex with men in Nigeria. Afr J Reprod Health 16: 21–28. 23444540

[6] Vu L, Tun W, Sheehy M, Nel D (2012) Levels and correlates of internalized homophobia among men who have sex with men in Pretoria, South Africa. AIDS Behav 16: 717–723. 21484279 10.1007/s10461-011-9948-4

[7] Longfield K, Astatke H, Smith R, McPeak G, Ayers J (2007) Men who have sex with men in Southeastern Europe: Underground and at increased risk for HIV/STIs. Cult Health Sex 9: 473–487. 17687673 10.1080/13691050601161864

[8] Laurent E (2005) Sexuality and human rights: an Asian perspective. J Homosex 48: 163–225. 15814505 10.1300/J082v48n03_09

[9] Logie CH, Newman PA, Chakrapani V, Shunmugam M (2012) Adapting the minority stress model: associations between gender non-conformity stigma, HIV-related stigma and depression among men who have sex with men in South India. Soc Sci Med 74: 1261–1268. 22401646 10.1016/j.socscimed.2012.01.008

[10] Gilmour S, Li J, Shibuya K (2012) Projecting HIV transmission in Japan. PLoS ONE 7(8): e43473 doi: 10.1371/journal.pone.0043473 22916268 10.1371/journal.pone.0043473 PMC3423344

[11] Hidaka Y, Ichikawa S, Koyano J, Urao M, Yasuo T, et al (2006) Substance use and sexual behaviours of Japanese men who have sex with men: a nationwide internet survey conducted in Japan. BMC Public Health 6: 239 doi: 10.1186/1471-2458-6-239 17002800 10.1186/1471-2458-6-239 PMC1599727

[12] DiStefano AS (2008) Suicidality and self-harm among sexual minorities in Japan. Qual Health Res 18: 1429–1441. 18689528 10.1177/1049732308322605

[13] Hidaka Y, Operario D (2006) Attempted suicide, psychological health and exposure to harassment among Japanese homosexual, bisexual or other men questioning their sexual orientation recruited via the internet. J Epidemiol Community Health 60: 962–967. 17053285 10.1136/jech.2005.045336 PMC2465476

[14] Asthana S, Oostvogels R (2001) The social construction of male ‘homosexuality’ in India: implications for HIV transmission and prevention. Soc Sci Med 52: 707–721. 11218175 10.1016/s0277-9536(00)00167-2

[15] Choi KH, Zhen N, Gregorich S, Pan QC (2007) The influence of social and sexual networks in the spread of HIV and syphilis among men who have sex with men in Shanghai, China. J Acquir Immune Defic Syndr 45: 77–84. 17325608 10.1097/QAI.0b013e3180415dd7

[16] Deuba K, Ekström AM, Shrestha R, Ionita G, Bhatta L, et al (2013) Psychosocial health problems associated with increased HIV risk behavior among men who have sex with men in Nepal: a cross-sectional survey. PLoS One 8(3): e58099. 23516434 10.1371/journal.pone.0058099 PMC3596342

[17] Latypov A, Rhodes T, Reynolds L (2013) Prohibition, stigma and violence against men who have sex with men: effects on HIV in Central Asia. Central Asian Survey 32: 52–65.

[18] Meyer IH (2003) Prejudice, social stress, and mental health in lesbian, gay, and bisexual populations: conceptual issues and research evidence. Psychol Bull 129: 674–697. 12956539 10.1037/0033-2909.129.5.674 PMC2072932

[19] Waldner-Haugrud LK, Magruder B (1995) Male and female sexual victimization in dating relationships: gender differences in coercion techniques and outcomes. Violence Vict 10: 203–215. 8777187

[20] Lloyd S, Operario D (2012) HIV risk among men who have sex with men who have experienced childhood sexual abuse: systematic review and meta-analysis. AIDS Educ Prev 24: 228–241. 22676462 10.1521/aeap.2012.24.3.228

[21] Fergusson DM, McLeod GF, Horwood LJ (2013) Childhood sexual abuse and adult developmental outcomes: findings from a 30-year longitudinal study in New Zealand. Child Abuse Negl. doi: 10.1016/j.chiabu.2013.03.013. [Epub ahead of print]. 10.1016/j.chiabu.2013.03.013 23623446

[22] Maman S, Campbell J, Sweat MD, Gielen AC (2000) The intersections of HIV and violence: directions for future research and interventions. Soc Sci Med 50: 459–478. 10641800 10.1016/s0277-9536(99)00270-1

[23] Birnbaum MH (2004) Human research and data collection via the Internet. Ann Rev Psychol 55: 803–32. 14744235 10.1146/annurev.psych.55.090902.141601

[24] Rhodes SD, Bowie DA, Hergenrather KC (2003) Collecting behavioural data using the world wide web: considerations for researchers. J Epidemiol Community Health 55: 68–73. 10.1136/jech.57.1.68 PMC1732282 12490652

[25] Radloff LS (1977) The CES-D scale: a self report depression scale for research in the general population. Appl Psychol Meas 1: 385–401.

[26] Hosmer DW, Lemeshow S (2013) Applied logistic regression. New York: Wiley. 12p.

[27] Shimane T, Hidaka Y, Matsuzaki Y (2012) Study via the internet about HIV preventive behavior among MSM. In: Annual report of a research group for Grant-in-Aid for AIDS Research from the Ministry of Health, Labour and Welfare of Japan “Intervention research by monitoring survey and cognitive behavior theory via the internet targeting populations at risk of HIV, and research on creating support system by various professional of human support services”. pp. 127–249. (in Japanese).

